# Sialic acid metabolism as a potential therapeutic target of atherosclerosis

**DOI:** 10.1186/s12944-019-1113-5

**Published:** 2019-09-14

**Authors:** Chao Zhang, Jingyuan Chen, Yuhao Liu, Danyan Xu

**Affiliations:** 1 Department of Cardiovascular Medicine, The Second Xiangya Hospital, Central South University, 139 Middle Renmin Road, Changsha, 410011 Hunan China; 2 Department of Health Management Center, Hunan Provincial People’s Hospital, 61 Jiefang West Road, Changsha, 410005 Hunan China

**Keywords:** Atherosclerosis, Sialic acid, Sialidase, Sialyltransferase, Trans-sialidase

## Abstract

Sialic acid (Sia), the acylated derivative of the nine-carbon sugar neuraminic acid, is a terminal component of the oligosaccharide chains of many glycoproteins and glycolipids. In light of its important biological and pathological functions, the relationship between Sia and coronary artery disease (CAD) has been drawing great attentions recently. Large-scale epidemiological surveys have uncovered a positive correlation between plasma total Sia and CAD risk. Further research demonstrated that N-Acetyl-Neuraminic Acid, acting as a signaling molecule, triggered myocardial injury via activation of Rho/ROCK-JNK/ERK signaling pathway both in vitro and in vivo. Moreover, there were some evidences showing that the aberrant sialylation of low-density lipoprotein, low-density lipoprotein receptor and blood cells was involved in the pathological process of atherosclerosis. Significantly, the Sia regulates immune response by binding to sialic acid-binding immunoglobulin-like lectin (Siglecs). The Sia-Siglecs axis is involved in the immune inflammation of atherosclerosis. The generation of Sia and sialylation of glycoconjugate both depend on many enzymes, such as sialidase, sialyltransferase and trans-sialidase. Abnormal activation or level of these enzymes associated with atherosclerosis, and inhibitors of them might be new CAD treatments. In this review, we focus on summarizing current understanding of Sia metabolism and of its relevance to atherosclerosis.

## Introduction

Coronary artery disease (CAD) arising from atherosclerosis is currently one of the leading causes of death worldwide [1]. Despite rapid advances in understanding the onset and progression of atherosclerosis, there is still limited life-saving drugs and it is of great need to develop new strategies for CAD treatment. In recent years, advances in metabolomics have improved our knowledge on the relationship between CAD and many metabolites, among which sialic acid (Sia) is one of the most critical ones.

**Fig. 1:**
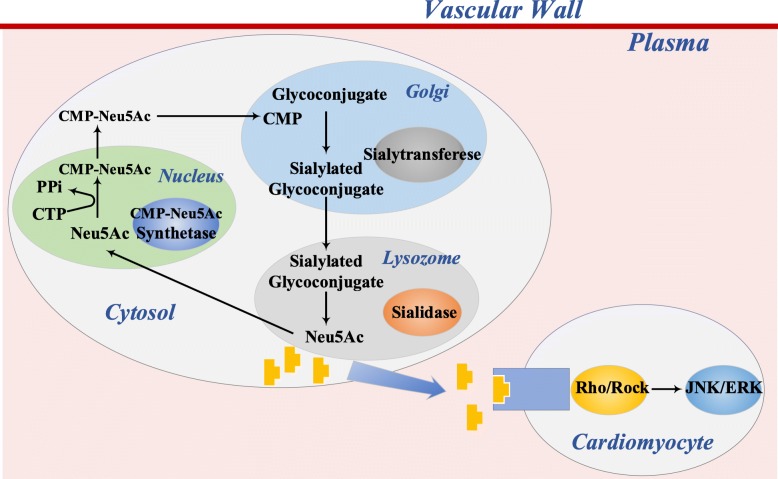
The summary of mammalian Sia metabolism and the Neu5Ac-activated signaling pathway in cardiomyocyte. Legend: In eukaryotic cells, Neu5Ac is synthesized in cytoplasm and transferred to nucleus for cytosine 5′-monophosphate (CMP)-Neu5Ac synthesis by CMP-Neu5Ac activating acid. Then, it is transmitted to Golgi apparatus to form glycoconjugates by ST, which is subsequently secreted or delivered to cell surface. N-Acetyl-Neuraminic Acid (Neu5Ac) as a signaling molecule to trigger myocardial injury via activation of Rho/ROCK-JNK/ERK signaling pathway in acute myocardial infarction

Sia is a family of monosaccharides with nine-carbon backbone and has high structural diversity. Sia always occupies the terminal position of the oligosaccharide chains of many glycoproteins and glycolipids which plays pivotal roles in many important physiological and pathological processes, such as nervous system embryogenesis, cancer metastasis, bacterial and viral infection [2–5]. Clinical research has shown that high plasma total sialic acid (TSA) levels contributed to increased risk of future cardiovascular events independent of BMI, cholesterol and socioeconomic position [6]. Sia also associated with a number of risk factors of CAD, such as dyslipidemia, insulin resistance and immune responses. Latest study published in *Circulation* demonstrated that N-Acetyl-Neuraminic Acid (Neu5Ac), acting as a signaling molecule, triggered myocardial injury via activation of Rho/ROCK-JNK/ERK signaling pathway in acute myocardial infarction (AMI) mouse model, which was the first clarification of molecular mechanism of Sia in CAD [7] (Fig. 1). Actually, CAD patients have lower low-density lipoprotein (LDL) Sia content than normal individuals, indicating that LDL desialylation is an important step in the occurrence of atherosclerosis [8–10]. Besides, desialylated LDL is more inclined to get oxidative modification and accumulate than native LDL [11]. Sialic acid-binding immunoglobulin-like lectin (Siglecs), the receptor of Sia, has been well studied recently [12]. The Sia-Siglec axis of some immune cells including dendritic cells (DCs), treg cells, B lymphocytes and monocytes is closely related to the inflammation in atherosclerosis [13–15]. Enzymes in Sia metabolism, like sialidase (NEU), sialyltransferase (ST) and trans-sialidase (TS), are also involved in the process of atherosclerosis through different ways. Some inhibitors of these enzymes, such as oseltamivir and zanamivir, might have potential therapeutic implications for CAD. In light of this, we here to review the Sia in atherosclerosis and hope to provide new perspectives of treatment of CAD.

## Sia structure and metabolism

Sia is a large family of neuraminic acid derivatives with a nine-carbon backbone. More than 50 sialic acid forms have been found in nature, including the most abundant N-acetylneuraminic acid (Neu5Ac), non-humans N-glycolylneuraminic acid (Neu5Gc), 2-keto-3-deoxy-nonulosonic acid (or deaminoneuraminic acid) and their single or multiple O-acetyl derivatives [16]. The apparent absence of Neu5Gc in human body was recognized in 1973. Humans cannot synthesize endogenous Neu5Gc due to the loss of the ability to convert Neu5Ac to Neu5Gc. This biochemical reaction is catalyzed by the enzyme cytidine monophosphate N-acetyl-D-neuraminic acid hydroxylase (CMAH) in all other mammals, while the inactivating mutation of the CMAH gene in humans results in the aberrant expression of a nonfunctional enzyme. Therefore, some researches indicated that normal metabolic incorporation of the non-humans Neu5Gc from dietary sources (mainly red meat) to humans tissues (mainly endothelia and epithelia) could induce the circulating anti-Neu5Gc antibodies, and then lead to chronic inflammation, such as atherosclerosis [17–19]. For the purposes of this manuscript, the term “Sia” will refer to Neu5Ac.

The metabolism of Sia is complex. In eukaryotic cells, Neu5Ac is synthesized in cytoplasm and transferred to nucleus for cytosine 5′-monophosphate (CMP)-Neu5Ac synthesis. Then, it is transmitted to Golgi apparatus to form glycoconjugates by ST, which is subsequently secreted or delivered to cell surface [20] (Fig. 1). Sia is usually located at the end of the oligosaccharide chain of glycoproteins and glycolipids, with a rarely free unit [21]. They are widely distributed in various fluids of human body (blood plasma, breast milk, bile, sweat, gastric juice, urine) and tissues (salivary glands, stomach, intestines, cartilage, etc.) In plasma, a large amount of Sia is present in orosomucoid, ceruloplasmin, fibrinogen, haptoglobin and in transferrin. They are also present in the glycoproteins of erythrocytes, leukocytes and platelets, particularly abundant in vascular endothelial cell surface.

Sia is monosaccharide that frequently terminates glycan structures. Due to their terminal position and properties, Sia can participate directly or indirectly in multiple cellular events and overall immune response. Sia could stabilize conformational of molecules by providing negative charges and proteolytic cleavage resistance, which are responsible for cells’ normal physiological function [22]. Interestingly, Sia can function as biological mask or as recognizable cell patterns. In the former way, Sia acts as anti-recognition agent by masking recognition sites such as polysaccharide of glycan chains or proteins on cell membranes, including receptor molecules. In this way, Sia helps shield host cells from pathogen recognition and contributes to cells being “self” and thus weakens immunoreactivity. In the opposite way, Sia also plays a role as a ligand for a variety of molecules, such as lectins, antibodies, hormones and bacteria, mycoplasma, viruses. Sia can aid cell recognition, adhesion and signal transferring [23]. In particular, the Sia-Siglecs axis has recently been recognized as being involved in most important phenomena of cellular and molecular interactions in immune regulation [12, 24]. In this respect, Sia has been associated with inflammatory diseases, malignancies, cardiovascular disease and diabetes.

## Sia and CAD

### TSA and CAD

The normal range of human plasma TSA (bound and free) is 1.58–2.22 mmol/L, of which glycoprotein Sia accounts for more than 70%, with small amount of free Sia (0.5–3 μmol/L). Substantial evidences have shown that TSA was a possible indicator of atherosclerosis and cardiovascular diseases in the general population. Lindberg et al. conducted a 20-year follow-up study of 26,693 men and 27,692 women in Sweden, and results showed that high TSA level was a risk factor of CAD, where men and women had a relative risk of death from CAD of 2.38 and 2.62 respectively, compared with death from non-cardiovascular diseases [25]. A large population-based cohort study, following participants for more than 35 years, showed that elevated plasma TSA level contributed to increased risk of cardiovascular events independent of BMI, cholesterol and socioeconomic position [26]. The increase of TSA might reflect the existence or progress of atherosclerosis. Gokmen et al. compared TSA level of 90 patients that were divided into three subgroups, which were with no vessel disease, with single-vessel disease, and with double/triple-vessel disease [27]. Patients with vessel disease had obviously increased TSA level compared to subjects with no vessel disease. Meanwhile, the number of diseased coronary arteries increased as TSA level increased. This positively correlative relationship between plasma TSA level and atherosclerosis severity has also been confirmed by many other studies. Patients with AMI or unstable angina pectoris (UAP) had much higher TSA level than those with stable angina pectoris (SAP) and normal coronary artery. However, Salomone and others argued that there was no difference between CAD patients and healthy individuals in terms of serum TSA level [28, 29]. Moreover, Govindarajan and colleagues showed that TSA level was related with complications of CAD rather than with CAD severity. For example, compared to AMI patients without any complications, patients with AMI which was accompanied by heart failure or arrhythmia had significantly higher TSA level. The different findings might be attributed to the different experimental or statistical methods. Hence more studies are needed to clarify the relationship between TSA level and CAD.

The reasons for increased plasma TSA are inconclusive. The released Sia from the cell surface is considered to be the main source [3, 21]. The increased output of serum acute-phase proteins by the liver caused by acute-phase reaction is another reason since many of the acute-phase proteins are glycoproteins with Sia residues.

### Lipoprotein sialic acid (LSA), lipid profile and LDL metabolism

The normal range of human serum LSA is only 10–50 μmol/L. Plasma very low–density lipoprotein (VLDL), LDL, intermediate-density lipoprotein (IDL), high-density lipoprotein (HDL), and lipoprotein(a) are sialylated at their apolipoprotein and glycolipid constituents. Sia preferentially binds ApoB100, ApoA, ApoE, Apo (a), and ApoC. The content of plasma LSA could differ considerably as a result of (1) variations in the sialylation of apolipoproteins before their secretion into plasma; (2) variations in the amount of sialic acid-containing apolipoproteins on lipoprotein in plasma; (3) modifications of the Sia on lipoprotein constituents following their secretion in plasma [30–32]. The LSA is associated with the charge of lipoproteins, lipoprotein solubility, receptor binding and uptake, cholesterol efflux and interactions with vascular matrix.

The serum concentration of Sia is also affected by serum lipids. A research with 382 healthy subjects indicated that serum Sia level was significantly higher in groups with high concentrations of triglyceride or total cholesterol while significantly lower in a group with high concentrations of HDL [33]. Accordingly, a study with 200 consecutive healthy subjects suggested that Sia had a remarkable negative correlation with serum HDL and an obvious positive correlation with serum triglycerides [34]. All these are predictors of atherosclerosis. Oppositely, the study showed that serum Sia had a non-significant correlation with total cholesterol and LDL. The research between Sia and LDL are further developed. As the core lipoprotein in the process of atherosclerosis, LDL contains 11 μg Sia per mg protein which affects its secretion, clearance, binding and uptake. Clinical studies have found that LDL from CAD patients, with 40–75% lower Sia level than healthy individuals, possessed a greater propensity of atherogenic [35]. Specifically, the binding ability of desialylated LDL, such as following combination with LDL receptor (LDLR), scavenger receptors, asialoglycoprotein receptors (ASGPR), or arterial proteoglycans, which was 1.5–2 times higher than that of native LDL [25, 36]. Meanwhile, the ability of desialylated LDL to induce cholesterol accumulation by arterial wall smooth muscle cells in humans was 2–4 times higher than that of native one [37]. Furthermore, Cerne et al. demonstrated that decreased Sia content of LDL was associated with an increased fraction of oxidized LDL. Desialylated lipoproteins were more susceptible to oxidative modification [38, 39]. Tertov proved that LDL desialylation was a primary step in atherogenic modification in humans plasma [9]. Therefore, preventing LDL desialylation might be a key step in anti-atherosclerosis treatment.

The clearance of 65–70% of LDL relies on LDLR on the surface of hepatocytes. As an integral glycoprotein, LDLR ends with Sia residues, which is linked with LDLR stability. Desialylated LDLR was less stable than sialylated LDLR. Asialoglycoprotein receptor (ASGPR) could interact with desialylated LDLR, leading to LDLR degradation [40]. In addition, LDL is also cleared through receptors on vascular endothelial cells, macrophages and arterial wall smooth muscle cells. Some studies have regarded that Sia formed a protective barrier against neointimal development. Even slight and presumably transient reduction of arterial Sia could trigger a sequence of events that lead to intimal thickening. The phenomenon was mainly caused by decreased anion density of endothelial cell surfaces after the removal of Sia. In this case, it promoted arterial endothelial cell uptake of LDL and fibrinogen, then stimulated the proliferation of smooth muscle cells in the arterial intima [38]. Nao Matsuka has showed that oxidatively modified Sia of LDL might be a probable binding site recognized by macrophages [41]. Thus, it may be important to investigate the role of Sia as a controlling factor in the catabolism of LDL.

### Sia and insulin resistance

Sia plays multiple roles in the control of glucose-transport activity. Many studies have shown that Sia level are associated with insulin resistance (IR). Epidemiology research has shown a significant increase in TSA level in patients with impaired glucose tolerance or newly diagnosed type 2 diabetes [42]. It is unclear why patients with IR or choronic hyperglycemia have higher serum TSA level. Studies have shown that the Sia synthesis was stimulated in liver and kidney under hyperglycemia [43]. Insulin receptor is a highly glycosylated membrane protein receptor and Sia is an essential for the function of the insulin receptor. Thus, it’s reasonable to increase the functional insulin receptor by up-regulating the synthesis of Sia under IR. Multiple studies have established that sialylation and desialylation constitutes a novel type of modulation of activity of cellular receptors including insulin receptor. Studies have confirmed that NEU1 activated the insulin receptor signaling by desialylating it. Pretreatment of adipocytes with NEU1 resulted in an increase in basal glucose transport. So Sia is closely related to insulin receptor [44].

Skeletal muscle is the main target organ of insulin, in which up to 80% of whole-body glucose disposal normally occurs. Impaired insulin reaction with a diminution in skeletal muscle insulin delivery is a major aspect of obesity-induced insulin resistance [45, 46]. Further research demonstrated that the inhibitory IgG FcγRIIB in skeletal muscle vascular endothelial cells played a key role of obesity-induced insulin resistance. IgG FcγRIIB reduced insulin transcytosis of endothelial cells by binding to desialylated IgG [47]. The desialylated IgG increased FcγRIIB affinity by 10-fold [48]. Research has shown that the Sia level of IgG in patients with type 2 diabetes (T2DM) was significantly lower than in healthy controls. Accordingly, IgG transferred from T2DM patients but not from metabolically healthy subjects resulted in insulin resistance in IgG-deficient mice via binding to FcγRIIB, indicating that similar processes may be applicative in T2DM in humans. Further studies indicated that the sialylation of IgG prevented insulin resistance. In mice fed with high-fat diet (HFD), supplementation with the Sia precursor N-acetyl-D-mannosamine restores IgG sialylation and maintained insulin sensitivity without affecting weight gain [49]. This is consistent with another study that exogenous Sia supplementation ameliorated the HFD-induced insulin resistance. The results showed that low and high doses of Sia improved metabolic indices, for example, the oral glucose tolerance test, serum TG, leptin, and adiponectin were significantly better than those in the HFD and HFD+ simvastatin groups [50]. The study demonstrated that Sia prevented HFD-induced insulin resistance through transcriptional and nontranscriptional mechanisms. At lower doses, sialylation of glycoproteins may be responsible for the protective effect. At higher doses, Sia induced transcriptional regulation of insulin signaling genes that can provide a long-lasting effect. Specifically, it upregulated the expression of Phosphoinositide-3-kinase, a central mediator of the intracellular signal transduction of insulin sensing, whose transcriptional downregulation has been related to obesity induced IR [51].

### The Sia-Siglecs axis of immune cells in CAD

Siglecs are negative regulators of immune cells, including dendritic cells (DC), treg cells, B lymphocytes and monocytes. Usually, Sia acts as a self-related molecular pattern, so Siglecs can act as a “self” sensor to protect the overreaction of immune system [52]. Most Siglecs, except for Siglec-1, have an intracellular immunoreceptor tyrosine-based inhibition motif that mediates inhibitory signals [53]. Siglecs have distinct binding preferences for the type of linkage and modifications of Sia. The immune cells expressing Siglecs can bind to Sia or glycoproteins present on another cell, which is called trans interaction. Siglecs can also bind to Sia exposed on the same cell, called cis interaction [54]. Although the biological functions of many Siglecs are largely unknown, Siglecs have been recognized as important immuno-modulatory receptors that alter immune cell function by binding to Sia. The interference in the Sia-Siglec axis is central in the balance between immune responses and immune tolerance. This concept is further supported by recent studies showing that disorders in the Sia-Siglec axis contribute to inflammation, autoimmunity, infection, aging and cancer.

#### The Sia-Siglecs axis of B-1 cells in CAD

It has been acknowledged in recent research that atherosclerosis is a chronic vascular inflammation which initiated and maintained by the generation of oxidized LDL (OxLDL) and other immune cells [55]. The interaction between inflammation and atherosclerosis development is partially mediated by the Sia-Siglec axis of immune cells. As a subset of lymphoid B cells, B-1 cells are emerging players in the chronic inflammation of metabolic diseases, such as obesity, diabetes, and atherosclerosis [56]. B-1 cells exhibit their atheroprotective effects via the secretion of low-affinity IgM antibodies [57]. Epidemiological research in humans demonstrated that high levels of OxLDL-specific IgM were connected with a lower risk of cardiovascular disease [56]. Siglec-G is mainly expressed on B-1 cells. Studies have shown that Siglec-G promoted atherosclerosis and liver inflammation by inhibiting the protective function of B-1 cells [13]. In Siglec-G-deficient LDLR−/− mice, there were increased B-1 cell-derived IgM that exceeded physiological plasma levels, and IgM inhibited the proinflammatory properties of OxLDL. Concretely, the OxLDL-specific IgM could inhibit foam cell formation by blocking scavenger receptor-mediated uptake of OxLDL by macrophages. Furthermore, anti-OxLDL IgM could prevent the accumulation of apoptotic cells by accelerating their uptake by macrophages [58, 59]. Simultaneously, the research identified an anti-inflammatory effect of Siglec-G deficiency in cholesterol-fed LDLR−/− mice as documented by reduced plasma levels of adhesion molecules, pro-inflammatory cytokines and chemical factors, and inflammatory mediator CXC chemokine receptor 1 (CXCL1) were also significantly reduced. CXCL1 is a key mediator of leukocyte recruitment in atherosclerosis [60]. The pathogenic role of Siglec-G in atherosclerosis has been demonstrated, it may represent a therapeutic approach to enhance endogenous defense mechanisms to prevent cardiovascular disease through blocking the inhibition of Siglec-G.

#### The Sia-Siglecs axis of DCs in CAD

Antigen-presenting cells, predominantly DCs, are critical to maintain the balance of immune system, because these cells determine whether an immune response will be initiated against pathogens or self or innocuous foreign antigens. Sia-modified antigens alter their immunogenicity. In the inflammatory response, the sialylated antigen inhibited immunization by binding to Siglec-E of DCs in an antigen-specific tolerogenic manner [61]. Specifically, it increased the number of Treg cells and inhibited the expansion of effector T cells and the generation IFN-γ. Treg cells are immunoregulatory cells, whose most important role is to maintain immune tolerance to self and harmless exogenous antigens. Studies have shown that treg cells played protective roles in atherosclerosis, while Treg cells were reduced in patients with coronary atherosclerosis [62, 63]. Thus, sialylation of specific antigens such as OxLDL may be a novel approach to improve atherosclerosis by modulating the effects of DC cells on Treg cells and effector T cells.

#### The Sia-Siglecs axis of macrophages in CAD

Siglec-1 (sialoadhesin, CD169) is highly expressed on circulating monocytes and plaque macrophages in atherosclerotic patients and may be considered as a potential risk marker for CAD [15, 64]. The positive rate of monocytes expressing Siglec-1 in CAD group was significantly higher than that in healthy controls [(11.5 ± 3.9)% versus (1.8 ± 2.0)%, P<0.01], but no significant differences were found among different CAD groups (AMI, SA, UA) or between CAD patients with normal level of serum lipids and abnormal level of serum lipids. Moreover, Siglec-1 may be considered as a potential non-invasive indicator for monitoring disease severity [15].

Siglec-1 lacks tyrosine-based signaling motifs and its cytoplasmic tail is poorly conserved, which suggests the primary role for Siglec-1 is as a binding partner in cell-to-cell interactions, rather than participating in cell signaling. Siglec-1-positive macrophages participate in the process of endothelial cells adhesion, lipid internalization, antigen presentation and pro-inflammatory cytokines secretion. The study demonstrated that blockade of Siglec-1 by lentivirus-mediated siRNA can prevent atherosclerotic lesion formation and proinflammatory cytokines (IL-1, IL-6, TNF-α and IL-17) production in Apoe−/− mice. Inhibition of Siglec-1 can diminish monocytes adhere to vascular endothelial cells via MCP-1/CCR2 and CXCL2/CXCR2 axis [65]. Futhermore, there are lower plaque macrophages accumulation and OxLDL uptake in the early process of atherosclerosis in Lv-shSiglec-1 mice. More importantly, increased Siglec-1 expression on monocytes was related to the increased T cell proliferation in CAD patients. However, down-regulation of Siglec-1 could attenuate proliferation and activation of cocultured T effector cells [14]. In summary, Siglec-1 can promote chemokines and pro-inflammatory cytokines secretion and influence the inflammatory process of atherosclerosis. Thus, pharmacological approaches targeting Siglec-1 may prevent disease progression and shed new light on the treatment of atherosclerosis.

### Sia of erythrocyte, platelet and leukocyte in CAD

Sia can also be found in erythrocyte and platelet membrane glycoproteins and is associated with their lifespan. Glycoprotein-bound Sia of erythrocyte and platelet could mask the antigen or the specific recognition sites on the cell surface, while decreased Sia residues could exposure a well-established signal. Consequently, the desialylated erythrocyte and platelet could be recognized and eliminated from the bloodstream by binding with the specific receptors in the liver. In fact, the erythrocyte and platelet surface Sia content decreased in patients with AMI, leading to an increased cell adhesion index and formation of thrombosis [66].

Atherosclerosis in part is an inflammatory disease. Leukocyte plays a crucial role in the inception, progression and complications of atherosclerosis, and its accumulation within the vascular wall is a major feature. Sia of leukocyte promoted the process of atherosclerosis, which was the opposite of that in erythrocyte and platelet. Leukocyte chemotaxis depended on the surface Sia derivative (sialylLewis X) and E-selectin of vascular endothelial cells [67]. In tissue inflammation, cytokines stimulated endothelial cell production of E-selectin, which could recognize sialylLewis X on the leukocyte surface and bind it, promoting leukocyte adhesion to the vascular endothelium and subsequently to the inflammatory tissue.

## Enzymes in Sia metabolism and CAD

Three enzymes have the ability to remove or transfer sialic acid: (1) NEU, a glycoside hydrolase that can hydrolyze Sia from the glycoconjugate end; (2) ST, a family of glycosyltransferases involved in the biosynthesis of sialoglycoproteins and sialoglycolipids, which only uses CMP-Neu5Ac as a glycosyl donor; (3) TS, transfers Sia from the glycoconjugate end to the new glycoconjugate. These three enzymes are demonstrated to participate in the occurrence and development of atherosclerosis. Modulation or inhibition of these enzymes may prove useful for the development of novel therapeutic and diagnostic strategies for CAD.

### NEU and CAD

Four sialidases have been found in human cells. Besides the lysosomal sialidase NEU1, three additional humans sialidases, the cytosolic sialidase NEU2, the plasma membrane-associated sialidase NEU3 and the lysosomal or mitochondrial membrane-associated sialidase NEU4, have been identified. The content of NEU1 is the highest, while the content of NEU2 is extremely low and the levels of NEU3 and NEU4 are about 10% of NEUl in tissue separately. These four humans sialidases showed different substrate specificities and physiological functions. NEU1 is capable of hydrolyzing a wide range of glycoproteins, oligosaccharides and ganglioside near neutral pH. NEU2 and NEU4 can hydrolyze glycoproteins and gangliosides in neutral condition and weak acid condition respectively. NEU3 is a key enzyme for ganglioside hydrolysis [68]. So far, the relationship between NEU1 and CAD in these four enzymes is the most studied.

Currently, a study published in *Circulation* proved the abnormal activation of NEU1 in the heart of CAD patients, especially in AMI. NEU1 knockdown decreased plasma Neu5Ac level and ameliorated myocardial ischemia injury in vitro and in vivo, as evidenced by reductions in inflammatory cell accumulation and improvement of cardiac function. Notably, this protective effect was reversed by reintroduction of Neu5Ac into NEU1 deficient cells. Further study confirmed that Neu5Ac could bind with RhoA and Cdc42, and then activate Rho/ROCK-JNK/ERK apoptosis signaling pathway. The downstream concrete signaling molecular mechanisms were not clarified in this research [7].

NEU1 has also been demonstrated to be involved in coronary atherosclerosis by influencing lipid metabolism, inflammatory responses and insulin resistance. Yang and others have found lower non-HDL cholesterol in hypomorphic NEU1 mice, whose NEU1 activity significantly reduced compared with normal mice. Hypomorphic NEU1 expression led to increased LDLR stability by reducing endocytosis through human hepatocyte ASGPR. Besides, decreased level of proprotein convertase subtilisin/kexin 9 (PCSK9), which can bind to LDLR and target it for degradation, were observed in the hypomorphic NEU mice. Moreover, elevated cholesterol levels in the liver lowered the content of sterol response element binding protein-2 (SREBP-2), which is essential for the promoter of microsomal triglyceride transporter (necessary for VLDL synthesis). Consequently, there was higher LDL uptake and lower VLDL production in the liver, followed by the reduced CAD risk [69]. Directly, further research showed that hypomorphic NEU1 mice exhibit a significantly reduced atherosclerotic lesion size (> 50% reduction) in comparison with the Apoe−/− mice. In addition, the hypomorphic NEU1 mice displayed fewer macrophages, T cells, and smooth muscle cells in the aortic root compared with the Apoe−/− mice, implying a reduced degree of inflammation and cell recruitment within the plaque [70, 71]. In summary, NEU1 down-regulation could reduce non-HDL cholesterol, inhibit leukocyte transmigration and attenuate atherosclerosis in Apoe knockout mice.

Significantly, insulin receptor activation and insulin cell survival responses have been confirmed to be under the regulation of a membrane associated mammalian NEU1 [72]. In the current study, the Sia residues in the N-linked glycan chains of insulin receptor acted as key factors affecting its’ activity and insulin signaling. Neu1 desialylated and activated the receptor, thus providing a feedback mechanism for the regulation of glucose uptake. At a molecular level, insulin binding to insulin receptor rapidly induced its interaction with NEU1, which hydrolyzes Sia in the glycan chains of the receptor. The desialylated insulin receptor induced a conformational change that increased interaction between the receptor subunits in the dimer and caused activation associated with signal transmission. The inhibitor of NEU1, DANA blocked both interaction between the subunits and activation of the insulin receptor. Mice deficient in Neu1 rapidly developed glucose intolerance and insulin resistance after being fed with HFD. Indeed, the levels of insulin-induced phosphorylation of activated insulin receptor and AKT in livers and muscles of Neu1-deficient mice were significantly reduced as compared with that of control animals. However, the increase of NEU1 activity in insulin target tissues reversed insulin resistance and glucose intolerance. The further research uncoverd a novel Neu1 and matrix metalloproteinase-9 (MMP-9) cross-talk in alliance with neuromedin B G-protein coupled receptor (GPCR), which was essential for insulin-induced insulin receptor activation and cellular signaling [73]. All above, it is possible to view NEU1 as a new therapeutic target in insulin resistance.

Accumulative data manifested that Neu3 exerts a significant effect in cell growth, migration and differentiation [74]. Sung-Kwon et al. demonstrated that NEU3 overexpression inhibited matrix metalloproteinase-9 expression in vascular smooth muscle cells, suggesting that Neu3 could be an effective candidate for the prevention of vascular proliferative disorders in the early state of atherosclerosis. However, increased NEU3 activity may also contribute to plaque instability in atherosclerosis [75]. So, it seems a little difficult to apply the inhibitor of NEU3 to the experiment of CAD treatment.

### ST and CAD

ST is present in the endoplasmic reticulum and Golgi; it can transfer the sialic acid from CMP-Neu5Ac to the glycoprotein and glycolipid terminal chains. There are four classes of ST based on the type of glycosidic bond, namely ST3Gal I-VI, ST6Gal I-II, ST6GalNAc I-VI, and ST8Sia I-VI. Evidences have shown that ST played a dual role in the process of atherosclerosis through different mechanisms.

ST was involved in endothelial cell surface Sia re-synthesis after injury. Elena and colleagues confirmed that intima and plasma ST activity was increased in CAD compared to the control group [76, 77]. In vitro experiments, the level of cell surface Sia would immediately decrease when an endothelial cell was damaged, then rise after 24 h, corresponding to the increased ST content. Presumably, the increased ST activity in atherosclerotic intima and plasma is a self-protection mechanism for accelerating endothelial cell surface repair and delaying the development of atherosclerosis after damage. However, ST overexpression also promoted the formation of atherosclerosis by increasing inflammatory cell chemotaxis [78]. The chemokine receptor CXC chemokine receptor 2 (CXCR2) involved in chemokine-induced leukocyte adhesion and participated in mononuclear cell migration to atherosclerotic lesions [79]. St3Gal-IV significantly accelerated CXCR2-mediated leukocyte adhesion and macrophage transformation into foam cells by sialylating CXCR2 [80]. St3Gal-IV knockout reduced the range of atherosclerotic lesions and inflammatory cell content of atherosclerotic plaque in Apoe−/− mice on a high-fat diet in an obvious manner [78].

In summary, ST overexpression could promote the repair of arterial endothelial cells, but it also accelerated the formation of atherosclerosis by affecting the recruitment of inflammatory cells.

### TS and CAD

TS was first discovered in trypanosomes, and present in human serum and lipoprotein sub-components. There are three optimal pH levels for TS activity: 3.0, 5.0, and 7.0. Therefore, TS can be activated in the blood at neutral pH or play a role in inflammatory lesions when macrophages reduce the internal pH to 5.0.

In humans, LDL desialylation took place in the plasma. Informed research findings into the enzyme related LDL desialylation has been inconsistent and contradictory. Several groups have considered that NEU was responsible for this atherogenic modification of LDL [74, 81]. However, it is more believable that TS participated in the formation of atherosclerosis lesions by desialylating LDL. Studies have shown that the NEU inhibitor 2,3-dehydro-2-deoxy-2β-N-acetylneuraminic acid didn’t affect LDL desialylation, which supported the premise that TS, but not NEU, mediated LDL desialylation.

TS could remove Sia from VLDL, IDL and HDL particles. The rate of Sia transfered from these glycolipids is much lower than that from LDL [82]. TS was found more active in CAD patients. LDL desialylation was the first step in the process of LDL-induced atherosclerosis. Therefore, targeting TS for treatment might delay or even terminate the development of atherosclerosis.

## Perspectives

Just as a large number of clinical surveys have shown that plasma TSA was positively associated with mortality from CAD, it sounds reasonable to ameliorate atherosclerosis through reducing Sia level in vivo. Zhang et al. has demonstrated that two anti-influenza drugs, oseltamivir and zanamivir could lower Neu5Ac by suppressing NEU1 activity and inhibiting Rho/ROCK-JNK/ERK signaling pathway in the heart, then exert protective effects in AMI [7]. Clinically, cardiovascular patients diagnosed with influenza, the rate of recurrent vascular outcomes among the group treated with anti-influenza drugs such as oseltamivir was significantly lower relative to the untreated group [83, 84]. Therefore, targeting NEU1 by reducing Sia contents may represent a plausible therapeutic intervention for CAD treatment. Moreover, inhibition of NEU1 by the sialidase inhibitor 2-deoxy-2,3-didehydro-N-acetylneuraminic acid could decrease production of reactive oxygen species and reduce the inflammatory response [70, 85]. We conclude that NEU1 may represent a promising target for managing atherosclerosis. However, the application of ST and TS inhibitors in CAD is seldom.

It seems conflict to supplement exogenous Sia to improve the atherosclerosis. Actually, some studies have suggested that exogenous Neu5Ac might attenuate atherosclerotic lesion formation to some extent by targeting several mechanisms [86, 87]. Neu5Ac could 1) decrease triglyceride through activating lipoprotein lipase activity and lower cholesterol by accelerating reverse cholesterol transport [88]; 2) elevate antioxidant activity through scavenging reactive oxygen species and restoring the activity of antioxidant enzymes [88]; 3) relieve the inflammation by decreasing protein expressions of TNF-α [86]; 4) attenuate hypercoagulation on high fat diet-induced hyperlipidemic rats at the transcriptional level [89]; 5) replenishe diminished cell surface Sia by incorporating exogenous one into the humans cells [90]. Surprisingly, statin treatment could increase the Sia content of LDL in hypercholesterolemic patients [91]. The results from above studies might pave the way for the bioavailable Neu5Ac in the prevention of cardiovascular events.

## Conclusion

The relationship between Sia and CAD is complex. In CAD, increased NEU1 activity causes the cell surface desialylation and elevated free Neu5Ac, partially responsible for the rise of TSA. Subsequently, the plasma free Neu5Ac could trigger the myocardial injury by activation of Rho/ROCK-JNK/ERK signaling pathway. In that case, the ST activity would accordingly elevate to accelerate endothelial cell surface repair for ameliorating the atherosclerosis. Though current studies about these enzymes are not in-depth, the inhibitor of NEU1, such as oseltamivir and zanamivir might be potential antiatherosclerotic drugs in AMI through a lipid-independent pathological mechanism. Further investigations in vivo should be performed to better understand the efficacy of pharmacological intervention of NEU1 in UAP or SAP. Experimentally, it sounds helpful to add free Neu5Ac for CAD treatment through several aspects, while deeper research needs to verify whether exogenous Neu5Ac could trigger AMI just as mentioned above. It can be expected that understanding of the molecular and cellular mechanisms about the Sia metabolism might shed new light on curing atherosclerosis.

## Data Availability

Data sharing not applicable to this article as o datasets were generated or analysed during the current study.

## Abbreviations

AMI: Acute myocardial infarction
ASGPR: Scavenger receptors, asialoglycoprotein receptors
CAD: Coronary artery disease
CMAH: Cytidine monophosphate N-acetyl-D-neuraminic acid hydroxylase CMP cytosine 5′-monophosphate
CXCL1: CXC chemokine receptor 1
CXCR2: CXC chemokine receptor 2
DCs: Dendritic cells
HDL: High-density lipoprotein
HFD: High-fat diet
IDL: Intermediate-density lipoprotein
IR: Insulin resistance
LDL: Low-density lipoprotein
LDLR: LDL receptor
NEU: Sialidase
Neu5Ac: N-acetylneuraminic acid
Neu5Gc: N-glycolylneuraminic acid
OxLDL: Oxidized LDL
PCSK9: Proprotein convertase subtilisin kexin 9
SAP: Stable angina pectoris
Sia: Sialic acid
Siglecs: Sialic acid-binding immunoglobulin-like lectin
SREBP-2: Sterol response element binding protein-2
ST: Sialyltransferase
T2DM: Type 2 diabetes
TS: Trans-sialidase
TSA: Total sialic acid
UAP: Unstable angina pectoris
VLDL: Very low–density lipoprotein
